# Altered EEG Response of the Parietal Network in Asymptomatic *C9orf72* Carriers

**DOI:** 10.1002/hbm.70275

**Published:** 2025-07-25

**Authors:** Stefan Dukic, Kevin van Veenhuijzen, Henk‐Jan Westeneng, Roisin McMackin, Ruben P. A. van Eijk, Boudewijn T. H. M. Sleutjes, Bahman Nasseroleslami, Orla Hardiman, Leonard H. van den Berg

**Affiliations:** ^1^ Department of Neurology University Medical Center Utrecht Brain Center, Utrecht University Utrecht the Netherlands; ^2^ Academic Unit of Neurology School of Medicine, Trinity College Dublin, University of Dublin Dublin Ireland; ^3^ Discipline of Physiology School of Medicine, Trinity College Dublin, University of Dublin Dublin Ireland; ^4^ Biostatistics & Research Support Julius Center for Health Sciences and Primary Care, University Medical Center Utrecht Utrecht the Netherlands; ^5^ Department of Neurology Beaumont Hospital Dublin Ireland

**Keywords:** ALS, amyotrophic lateral sclerosis, asymptomatic, *C9orf72*, EEG, sustained attention to response task

## Abstract

Amyotrophic lateral sclerosis (ALS) is a neurodegenerative disease characterized by motor neuron degeneration. Around 10% of cases have a genetic basis, with the *C9orf72* hexanucleotide repeat expansion being the most common cause in individuals of European ancestry. Detecting early alterations in at‐risk individuals could aid in identifying biomarkers for timely diagnosis and intervention. In this study, we investigated electrophysiological changes in asymptomatic *C9orf72* mutation carriers using EEG, focusing on cognitive and motor networks, as these individuals are at risk of developing impairments in both domains. This study included 87 asymptomatic family members (AFM) of patients with familial *C9orf72* ALS, comprising 37 individuals carrying the pathological repeat expansion (C9+) and 50 without it (C9−). High‐density EEG was recorded during the sustained attention to response task (SART), which is a Go/NoGo paradigm that engages the frontoparietal and motor networks. Task performance was recorded and six behavioral measures were extracted: NoGo accuracy, Go accuracy, total accuracy, anticipation error, average response time, and response time variability. Analyses were conducted on EEG data in both sensor‐ and source‐space, using stimulus‐ and response‐locked data. The stimulus‐locked Go and NoGo data were analysed within two time windows: 180–350 ms (N2) and 300–600 ms (P3), while response‐locked Go data were analysed within a −100 to 100 ms time window. Linear mixed models were used to quantify differences between groups, incorporating familial pedigree to control for between‐subject dependencies. While the two groups did not significantly differ in any SART performance measures, EEG analyses revealed differences. During the stimulus‐locked N2, significant differences were observed in sensor‐space, primarily in central electrodes during both NoGo and Go conditions, with C9+ AFM exhibiting an increased negative potential. Source analysis confirmed these findings and localized the increased activity in the bilateral precuneus and superior parietal regions. Further analysis of the response‐locked data supported the involvement of the same posterior regions. No significant relationships were found between these EEG observations and SART performance. These findings provide the first evidence of EEG changes in AFM carrying the *C9orf72* repeat expansion. The observed functional changes in the parietal regions may reflect genotype‐related effects on the motor control network, potentially contributing to early pathophysiology. In contrast, clinical assessments and task performance did not differ between groups, suggesting that our EEG findings may hold promise as biomarkers for monitoring the risk of conversion to symptomatic disease and warrant further exploration to assess their predictive value for future symptom onset.

## Graphical abstract

Our study provides the first evidence of EEG changes in asymptomatic *C9orf72* repeat expansion carriers. Despite no clinical signs, these individuals exhibit altered activity in parietal regions associated with motor response control.

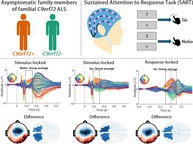

**Summary**

- This study is the first EEG investigation of asymptomatic family members of patients with familial *C9orf72* amyotrophic lateral sclerosis (ALS). It aimed to identify functional changes associated with *C9orf72* repeat expansion carriers using the sustained attention to response task (SART).
- Asymptomatic family members carrying the repeat expansion exhibited increased negative potential in central electrodes, localized to the bilateral precuneus and superior parietal regions.
- These findings suggest that increased activity in the parietal regions may reflect the subtle effects of the *C9orf72* genotype on the motor control network.

## 1 Introduction

Amyotrophic lateral sclerosis (ALS) is a progressive neurodegenerative disease characterized by the involvement of upper and lower motor neuron. While the majority of ALS cases are considered to occur sporadically, a notable subset of approximately 10% of patients of European ancestry have a familial form linked primarily to the GGGGCC hexanucleotide repeat expansion (RE) within the *C9orf72* gene (chromosome 9 open‐reading‐frame 72) (Veldink 2017). This mutation overlaps significantly with frontotemporal dementia (FTD) (Diekstra et al. 2014) and is now understood to be incompletely penetrant (Van Wijk et al. 2024). Early diagnosis of ALS remains a major challenge, with therapeutic outcomes likely dependent on timely intervention. Assessing individuals at risk during their presymptomatic phase could provide insights into subtle brain circuit dysfunction and help identify early biomarkers, potentially guiding the development and timing of therapies administered before irreversible damage occurs.

Although the presymptomatic stage of ALS is still not fully characterized, several research groups have shown structural changes in asymptomatic *C9orf72* RE carriers (Chipika et al. 2020). Most recently, our MRI study has shown grey matter changes, encompassing the motor cortex, as well as the parietal, occipital, and temporal regions (van Veenhuijzen et al. 2022). Besides structural changes, PET studies have recently produced evidence of glucose metabolism dysfunction in the frontotemporal regions, precuneus, basal ganglia, and thalamus (De Vocht et al. 2020; Popuri et al. 2021). In contrast, the neurophysiological changes in asymptomatic *C9orf72* RE carriers have not been elucidated. Research groups have, however, shown alterations in the motor cortex and frontoparietal networks in ALS using both EEG and MEG (Dukic et al. 2019; Nasseroleslami et al. 2019; Proudfoot, Colclough, et al. 2018a; Proudfoot, van Ede, et al. 2018b; Proudfoot et al. 2017). An EEG study (McMackin et al. 2020) using the sustained attention to response task (SART) (Robertson et al. 1997), a variation of a Go/NoGo task, showed increased engagement in the insula and the parietal cortex in ALS patients. By requiring both motor and cognitive performance, the SART is a highly suitable candidate for interrogating cohorts of those who are at risk of developing motor and cognitive impairments, such as *C9orf72* RE carriers.

Early detection of ALS is crucial, as the optimal therapeutic window likely occurs in the earliest stages of the disease, prior to observable clinical symptoms. A notable advantage of EEG is its ability to directly measure brain activity, enabling the detection of both network dysfunction and compensatory mechanisms. This positions EEG as a valuable tool for identifying early electrophysiological brain changes that may precede structural atrophy. Interestingly, a study on *C9orf72* RE carriers with FTD suggested that alterations in intracortical connectivity, as measured by transcranial magnetic stimulation, may occur before the onset of structural and cognitive changes (Benussi et al. 2019). Given its sensitivity to fast functional dynamics, noninvasive nature, cost‐effectiveness, and widespread availability, EEG is particularly suited for use as a biomarker to monitor disease progression or therapeutic response (Babiloni et al. 2021; Van Straaten et al. 2014).

As electrophysiological brain changes in asymptomatic *C9orf72* RE carriers remain largely unexplored, this study aimed to evaluate both cognitive and motor networks in this cohort using high‐density EEG. To engage these networks, we employed the SART paradigm, and to minimize the effects of intersubject genetic variation, we included only related asymptomatic individuals from families with a known familial history of ALS.

## 2 Methods

### 2.1 Participants

Between December 2020 and December 2024, we included 99 family members of patients with *C9orf72*‐associated familial ALS. All participants were recruited through relatives diagnosed with ALS at the Motor Neuron Disease Outpatient Clinic of the University Medical Center Utrecht. Participants were aged 18 years or older and provided written informed consent in accordance with the Declaration of Helsinki. This study was approved by the medical ethics committee of the University Medical Center Utrecht.

From the complete cohort of family members, three were excluded due to missing recent cognitive testing or neurological examination results, five due to abnormal cognitive test outcomes and therefore not considered asymptomatic, and four due to active use of psychoactive medication or a history of significant head trauma, which could confound electrophysiological findings. The remaining 87 participants were classified as asymptomatic family members (AFM), defined by the absence of clinical signs of upper or lower motor neuron disease, bulbar dysfunction, and cognitive impairment. Methods for determining asymptomatic status have been described previously (van Veenhuijzen et al. 2022).

Genetic testing determined the *C9orf72* RE status, with pathological expansions defined as ≥ 30 GGGGCC repeats in genomic DNA samples, as previously described (Van Rheenen et al. 2012). Using this criterion, we dichotomized the cohort into AFM with the pathological *C9orf72* RE (C9+) and those without it (C9−).

### 2.2 Experimental Paradigm

Participants completed the SART, where they were instructed to click the left mouse button each time a number appeared on the screen, except when the number “3” was displayed. The task was divided into 5‐min blocks, with appropriate breaks to minimize fatigue. Participants were seated approximately 1 m from a computer monitor, where single‐digit numbers (from one to nine) appeared randomly for 250 milliseconds (ms) using Presentation software (Neurobehavioral Systems Inc., Albany, CA). To reduce discomfort from bright light, digits were presented in light grey (RGB code: 250, 250, 250) on a black background. Font sizes were randomized between 100, 120, 140, 160, and 180 points to prevent participants from using a perceptual template for recognizing the number three and to promote cognitive processing of the numerical values. Each stimulus was followed by an interstimulus interval of 1120–1220 ms, during which a black screen was displayed. Each recording block contained 252 trials, with the number “3” appearing randomly in 11% of trials.

Participants were instructed to prioritize both speed and accuracy, as both served as performance measures. Under the supervision of the experimenter, each participant completed a practice round of up to 45 trials to ensure task comprehension. During the recording, the room lights were switched off to reduce visual distractions, and participants completed three or four blocks of the task.

### 2.3 Data Acquisition

The pedigree of all participants was documented, and each participant was tested for the pathogenic *C9orf72* hexanucleotide RE. All participants underwent a standardized, comprehensive neurological examination to rule out signs of upper and lower motor neuron involvement, as detailed in our prior study (Nitert et al. 2022). Additionally, abnormal cognitive status (defined as > 2 standard deviations from normative data) was assessed using the Dutch version of the Edinburgh Cognitive and Behavioral ALS Screen (ECAS), a screening tool designed to detect cognitive and behavioral changes specific to ALS (Bakker et al. 2019).

Task performance of the SART was recorded using Presentation software. High‐density EEG data with 128 electrodes were collected using the BioSemi Active Two system (BioSemi B.V., Amsterdam, The Netherlands), sampled at 512 Hz and lowpass filtered at 104 Hz by the acquisition hardware to prevent aliasing.

### 2.4 SART Data Analysis

Six behavioral measures were extracted: NoGo accuracy (percentage of three‐digit stimuli followed by response omission), Go accuracy (percentage of nonthree‐digit stimuli followed by a response within 170–650 ms), total accuracy (combined NoGo and Go accuracy), anticipation error (responses faster than 170 ms), median response time, and response time variability (calculated as the interquartile range of response times). All response times were retained to assess potential effects of fatigue (i.e., response time change over time). Response times and their derived measures were log‐transformed to address positive skew and approximate a normal distribution.

### 2.5 EEG Data Analysis

The analysis was conducted using custom automated MATLAB scripts, incorporating publicly available FieldTrip (Oostenveld et al. 2011), EEGLAB (Delorme and Makeig 2004), and RELAX (Bailey et al. 2023) toolboxes. Data were downsampled to 256 Hz, highpass filtered (noncausal Butterworth, 0.3 Hz cut‐off, 4th order) and 50 Hz line noise was removed using the Zapline‐plus method (Klug and Kloosterman 2022). Noisy electrodes were removed and interpolated using spherical spline interpolation (Perrin et al. 1989). Independent component analysis (Raimondo et al. 2012) was applied on common‐average referenced signals and 70 components were extracted, which were then checked for ocular, muscle and cardiac activity. Components representing ocular and cardiac activity were removed, while muscle‐related components were filtered (noncausal Butterworth, 15 Hz cut‐off, 4th order) to preserve low frequency neural activity.

Cleaned signals were then lowpass filtered (noncausal Butterworth, 60 Hz cut‐off, 4th order) and segmented into epochs ranging from −200 to 900 ms relative to stimulus event (stimulus‐locked dataset), and from −600 to 400 ms relative to response event (response‐locked dataset). Both datasets were then re‐referenced to the common average and baseline‐corrected by subtracting the mean amplitude of the baseline period: from −200 to 0 ms for the stimulus‐locked dataset, and from −600 to −400 ms for the response‐locked dataset. Trials were then screened for artefacts, and remaining ones were used for subsequent analysis. The preprocessing pipeline and the breakdown of the preprocessing outcomes and data quality assessments are detailed in the Supporting Information (see Note S1 and Table S1).

For the stimulus‐locked dataset, trials with incorrect task responses, multiple responses, responses within the baseline period, or response times shorter than 170 ms or longer than 650 ms were excluded from the subsequent event‐related potential (ERP) analysis. For the response‐locked dataset, used in the secondary analysis, only correct Go condition trials with a single response and response times between 170 and 400 ms were included in the analysis.

For both datasets, source localization was performed using the exact low resolution electromagnetic tomography (eLORETA) (Pascual‐Marqui, n.d.), with individualized, realistically shaped boundary element models based on each participant's T1‐weighted MRI scans (van Veenhuijzen et al. 2022). The MRI images were acquired using a 3T Philips Achieva Medical Scanner (Best, the Netherlands), with imaging parameters and preprocessing as previously described (Walhout et al. 2015). For eight participants who either did not have an MRI scan or whose models were of insufficient quality, a model based on the ICBM152 template (Fonov et al. 2009; Huang et al. 2019) was used. The models incorporated isotropic conductivity values for brain (0.33 S/m), skull (0.008 S/m), and scalp (0.43 S/m). A regular grid of dipoles spaced 5 mm apart (comprising 4298 dipoles representing grey matter) was used. For each participant, the diagonal values of the noise covariance matrix, estimated from baseline data, were used for whitening the leadfield. The signal‐to‐noise ratio, required for regularization in eLORETA, was set to three. Source signals were estimated for 68 cortical regions defined by the Desikan‐Killiany‐Tourville atlas (Klein and Tourville 2012). For each participant and brain region, an equivalent signal was estimated by extracting the strongest component of that region. This was achieved by applying singular value decomposition to the source‐space covariance matrix of that region. To resolve ambiguities in the polarity of the source time series across participants, singular value decomposition was applied on source‐space ERPs of all participants to orient the regional signals of each individual in the same direction.

For the stimulus‐locked dataset, group differences were assessed using EEG trials from each task condition (NoGo and Go) separately, based on the mean ERP amplitude within two time windows of interest: 180–350 ms (N2) and 300–600 ms (P3). These time windows were determined by visual inspection of the ERP waveforms and guided by previous research using the same paradigm (McMackin et al. 2020; Staub et al. 2015; Zordan et al. 2008). For the response‐locked dataset, group differences were assessed by comparing the mean Go ERP amplitude within a time window centered around the response (−100 to 100 ms).

### 2.6 Statistical Analysis

Differences in demographics between groups were assessed using the Mann–Whitney U test for continuous variables and Fisher's exact test for categorical variables.

Neuropsychological performance was compared using linear model analysis, with the ECAS outcome as the response variable and age, sex, education level, and *C9orf72* RE carriership as predictors. Upper motor neuron function was compared using binomial logistic regression for dichotomous outcomes, or ordinal regression for outcomes with more than two categories, with the assessment outcome as the response variable and age, sex, and *C9orf72* RE carriership as predictors. The most deviant neurological signs—whether on the left or right side—were included in the analysis.

Task performance measures were assessed as response variables using either linear models or beta regression analysis. Linear models were used for data that approximated a normal distribution (i.e., median response time and response time variability), while beta regression analysis was used for data naturally bounded between zero and one (i.e., measures of accuracy). The models included age, sex, and *C9orf72* RE carriership as fixed effects. Additionally, to assess potential effects of fatigue and group differences, a linear mixed‐effects model with response time as the response variable was used, with age, sex, and *C9orf72* mutation carriership included as fixed effects. To account for dependencies within the response time data, a random intercept for participant ID was included.

Group differences in ERP measures from both datasets (stimulus‐ and response‐locked) were evaluated using a linear mixed‐effects model, with age, sex, and *C9orf72* RE carriership included as fixed effects. To account for dependencies within the EEG data, a random intercept for participant ID was included, alongside a pedigree ID as an additional random effect to account for familial relationships. Separate models were fitted for each EEG channel and brain region. Multiple comparisons were controlled using the threshold‐free cluster enhancement (TFCE) method (E = 0.5, H = 2) (Mensen and Khatami 2013) with 5000 permutations, with neighbors using the radius of 40 mm. Statistical significance was set at *p* < 0.05, corrected for multiple comparisons. To minimize the computational demands of permutation testing, for the stimulus‐locked dataset, only correct Go trials immediately preceding correct NoGo trials were included in the analysis. This approach was deemed justifiable, as the raw *p* values obtained closely matched those derived from analyses using the full dataset.

We conducted two sensitivity analyses using sensor‐space stimulus‐locked data from both task conditions. First, to assess whether age differences influenced the observed ERP findings, we reanalysed data after excluding 11 C9‐AFM participants under the age of 30. This adjustment created a more comparable age distribution between groups (*p* = 0.6) while ensuring that other demographic and clinical confounders remained nonsignificant. The second sensitivity analysis excluded pedigrees with only one AFM (*N* = 16) to strengthen the assumption that pedigrees could be used as a control variable to account for familial relationships. In this analysis, potential confounders also did not differ significantly. As both sensitivity analyses yielded the same group differences, the main text presents only the results from the complete dataset (*N* = 87 AFM).

To investigate the relationship between ERP measures and SART performance, and the nature of observed differences in ERP measures, linear mixed‐effects models were employed. For each SART performance measure of interest, an initial model was specified with the ERP measure as the dependent variable, and age, sex, *C9orf72* RE carriership, and the SART measure as fixed effects. Similarly to the main analysis, random intercepts for each participant and pedigree were also included. This initial model assessed the overall ERP‐SART relationship, assuming a common association across cohorts. Subsequently, this model structure was extended by incorporating an interaction term between *C9orf72* RE carriership and the SART measure. This second modeling approach specifically tested whether the ERP‐SART association varied by *C9orf72* RE carriership status. *p* values were adjusted for multiple comparisons using the Benjamini‐Hochberg false discovery rate procedure (q = 0.05).

## 3 Results

### 3.1 The Demographic Profiles

In total, 87 AFM were analyzed, of which 37 were C9+ and 50 were C9−. The total number of pedigrees was 37, with a median number of AFM per pedigree: 2 (range: 1–13). All participants were up to the third degree of consanguinity with respect to the closest family member with ALS. Demographics of the participants are summarized in Table 1.

**TABLE 1:** Demographic and clinical characteristics of asymptomatic family members.

	AFM C9−	AFM C9+	*p*
*N*	50	37	
Age, year	41.4 (32.0–52.4)	49.5 (38.4–56.8)	0.04
Sex, male	22 (44)	19 (51)	0.52
Education level, high	30 (60)	24 (65)	0.81
Handedness, right	44 (88)	33 (89)	0.28
Pedigrees	37 (2; 1–13)		

*Note:* Data are shown as median (interquartile range) and count (%). Pedigree data are shown as total number of pedigrees (median number of AFM per pedigree; range). Education level was assessed using the Verhage education system and dichotomized into low (level 1–5) and high (level 6–7) level of education. *p* values were calculated using the Mann–Whitney U test for continuous variables and Fisher's exact test for categorical variables.

Abbreviations: AFM, asymptomatic family member; C9−, carriership of *C9orf72* with normal repeat length; C9+, carriership of *C9orf72* repeat expansion.

### 3.2 Physical Examination and Cognitive Functioning Scores Do Not Differ Between Groups

No clinical signs of lower motor neuron involvement (e.g., muscle atrophy, weakness, fasciculations, hyporeflexia) were detected during the physical examination. Mild signs suggestive of upper motor neuron involvement were observed in both groups, but no significant differences were found between them. Additionally, there were no significant group differences in cognitive functioning. A detailed breakdown of the physical examination and cognitive screening results is provided in Table S2.

### 3.3 Task Performance Is Similar Between Groups

The two groups did not differ significantly in any of the SART performance measures (Table 2), nor did they show different rates of fatigue (i.e., slowing of response times; *p* = 0.58).

**TABLE 2:** Performance measures of SART.

	AFM C9−	AFM C9+	*p*
Correct NoGo (%)	75 (60–83)	74 (64–83)	0.61
Correct Go (%)	97 (95–99)	97 (95–99)	0.81
Correct total (%)	95 (92–97)	95 (91–97)	0.99
Anticipation error (%)	1 (0–3)	1 (0–2)	0.78
Response time (ms)	290 (264–328)	298 (268–339)	0.69
Response time variability (ms)	84 (62–107)	80 (65–102)	0.15

*Note:* Data are shown as percentages (%) or milliseconds (ms) and presented as median (interquartile range). *p* values were calculated using beta regression analysis for data in percentages and linear model analysis otherwise.

Abbreviations: AFM, asymptomatic family member; C9−, carriership of *C9orf72* with a normal repeat length; C9+, carriership of *C9orf72* repeat expansion.

### 3.4 Sensor‐Space Stimulus‐Locked Analysis Shows Differences in the Central Electrodes

**FIGURE 1:**
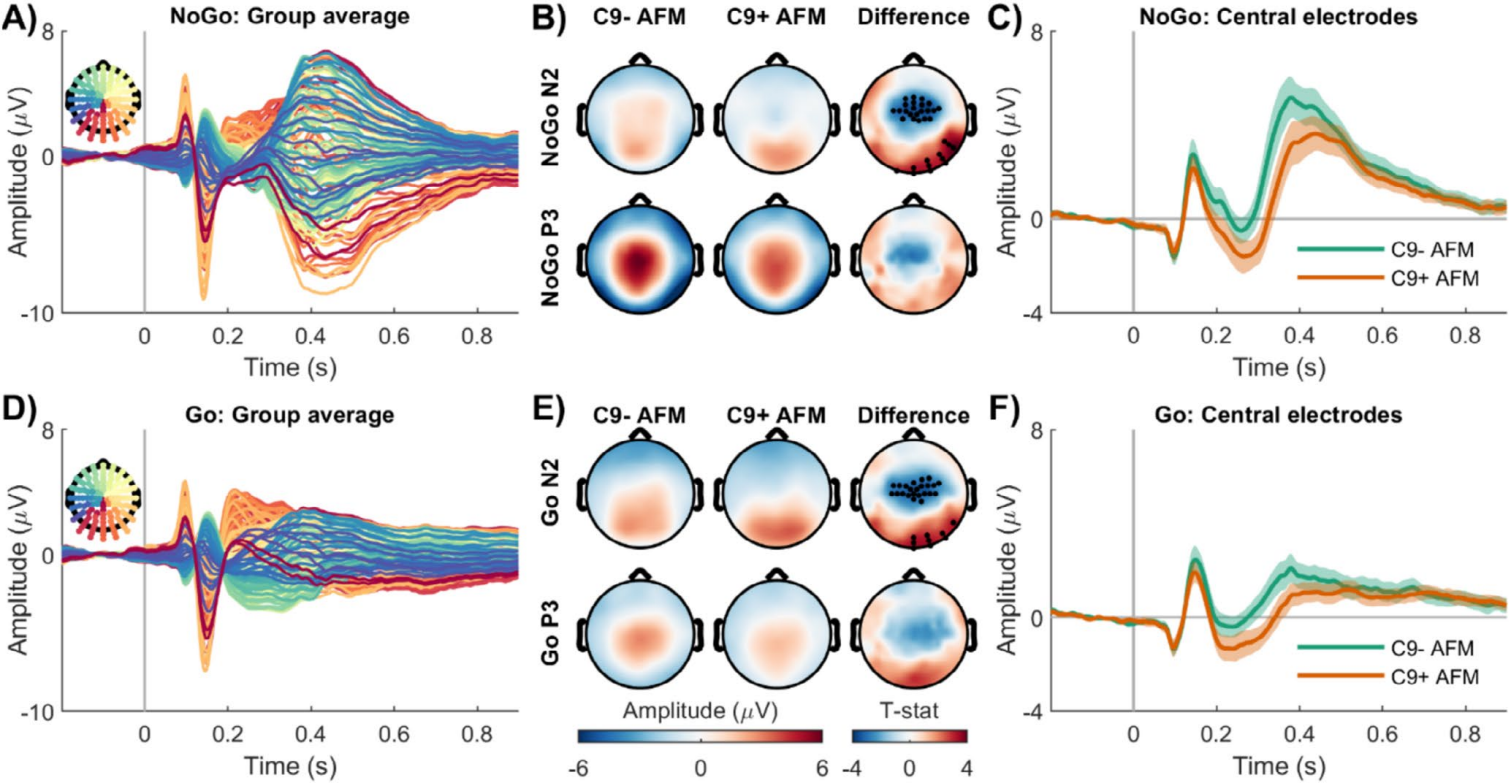
Differences in sensor‐space stimulus‐locked brain activations in asymptomatic *C9orf72* family members. (A) Group‐level stimulus‐locked average NoGo potentials for all family members. (B) Average NoGo N2 (180–350 ms) and P3 (300–600 ms) scalp potentials. During the N2, statistical analysis revealed significant group differences, primarily driven by a cluster of central electrodes, highlighted with bold black dots. During the P3, group differences were observed but did not remain statistically significant after correction for multiple comparisons. (C) The average potential from the significant central electrodes confirmed that this difference was due to an increased negative potential in C9+ AFM. (D) Group‐level average Go potentials for all family members. (E) Average Go N2 and P3 scalp potentials. During the N2, statistical analysis revealed significant group differences, again driven by a cluster of central electrodes (bold black dots). During the P3, group differences were observed but did not remain statistically significant after correction for multiple comparisons. (F) The average potential from the significant central electrodes confirmed that this difference was due to an increased negative potential in C9+ AFM. AFM, asymptomatic family members; C9−, carriership of *C9orf72* with normal repeat length; C9+, carriership of *C9orf72* repeat expansion.

The group‐level stimulus‐locked NoGo ERPs in sensor‐space for all family members is shown in Figure 1A (see also Figure S1). During the NoGo condition, within the N2 (180–350 ms), both cohorts exhibited positive potentials over the parietal and occipital electrodes (Figure 1B). In the C9− group, this positive potential extended toward the central electrodes. During the P3 (300–600 ms), both groups displayed a similar positive potential over centroparietal electrodes (Figure 1B). Statistical analysis revealed a significant difference between the two groups during the N2 only, primarily driven by a cluster of central electrodes, which appeared as a negative potential (Figure 1B). Further inspection of the average ERPs from these significant central electrodes confirmed that this difference was due to an increased negative potential in the C9+ AFM group (Figure 1C). The outcomes of the statistical models are provided in Table S3.

The group‐level stimulus‐locked Go ERPs in sensor‐space for all family members is shown in Figure 1D (see also Figure S1). During the N2, both cohorts displayed positive potentials over the parietal and occipital electrodes, while during the P3, both groups exhibited a positive potential over centroparietal electrodes (Figure 1E). Statistical analysis revealed a significant difference between the two groups during the N2 only, primarily driven by a cluster of central electrodes, which appeared as a negative potential (Figure 1E). Further inspection of the average ERPs from the significant central electrodes confirmed that this difference was due to an increased negative potential in the C9+ AFM group (Figure 1F). The outcomes of the statistical models are provided in Table S4.

### 3.5 Source‐Space Stimulus‐Locked Analysis Reveals Differences in the Parietal Regions

**FIGURE 2:**
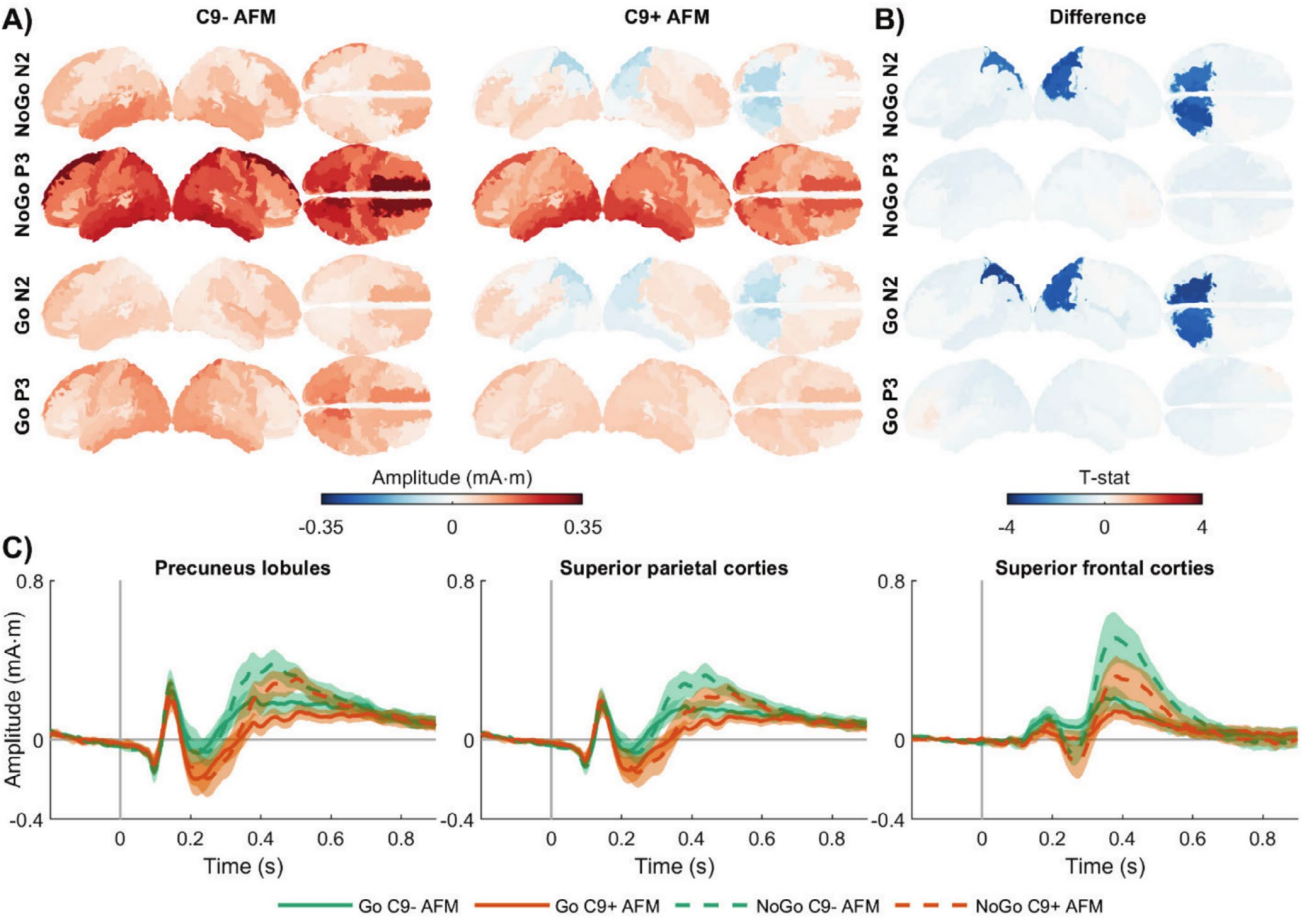
Differences in source‐space stimulus‐locked brain activations in asymptomatic *C9orf72* family members. (A) Group‐level stimulus‐locked average NoGo and Go potentials for each group during the N2 (180–350 ms) and P3 (300–600 ms) time windows. (B) A significant group difference was observed during the N2 in both task conditions, characterized by a more negative potential in the bilateral precuneus and parietal cortices in C9+ AFM. During the P3, group differences were observed in both task conditions but did not remain statistically significant after correction for multiple comparisons. (C) Average ERPs from these regions confirmed that this difference was driven by an increased negative potential in C9+ AFM. AFM, asymptomatic family members; C9−, carriership of *C9orf72* with normal repeat length; C9+, carriership of *C9orf72* repeat expansion.

The group‐level stimulus‐locked NoGo and Go ERPs in source‐space are shown in Figure 2A (see also Figure S2). Similar to the sensor‐level findings, both task conditions exhibited comparable activations, with those in the Go condition being less prominent than those in the NoGo condition. During the N2, the groups showed diverging activation patterns in the parietal and superior frontal cortices, but both exhibited activity in the middle frontal cortices. In contrast, during the P3, both groups displayed similar widespread brain activation, with primary generators located in the precuneus, motor, and superior frontal regions.

Statistical analysis revealed significant group differences during the N2 in both task conditions, in overlapping regions encompassing the bilateral precuneus, bilateral superior parietal, and right inferior parietal cortex (Figure 2B). The significance initially detected in the superior frontal gyri did not remain after correction for multiple comparisons. Further inspection of the average ERPs from these regions confirmed that these differences were driven by an increased negative potential in the C9+ AFM group (Figure 2C). The outcomes of the statistical models are reported in Tables S5 and S6.

A closer examination of the stimulus‐locked ERPs (Figures 1C,F and 2C) indicates that group differences span approximately the 200–400 ms time window poststimulus. As this time window overlaps with the interquartile range of response times (Table 2), we next analyzed the response‐locked data to determine whether these differences indeed pertain to motor response processes.

### 3.6 Response‐Locked Analysis Confirms the Involvement of the Parietal Regions

**FIGURE 3:**
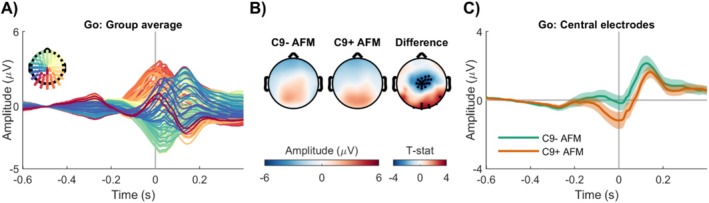
Differences in sensor‐space response‐locked brain activations in asymptomatic *C9orf72* family members. (A) Group‐level response‐locked average Go potentials for all family members. (B) Average scalp potentials from −100 to 100 ms around the response time. Statistical analysis revealed significant group differences, primarily driven by a cluster of central electrodes, highlighted with bold black dots. (C) The average potential from the significant central electrodes confirmed that this difference was driven by an increased negative potential in C9+ AFM. AFM, asymptomatic family members; C9−, carriership of *C9orf72* with normal repeat length; C9+, carriership of *C9orf72* repeat expansion.

The group‐level response‐locked Go ERPs in sensor‐space for all family members is shown in Figure 3A (see also Figure S3). Both groups exhibited activation patterns similar to those observed during the N2 in the stimulus‐locked dataset. Specifically, positive potentials were present over the parietal and occipital electrodes, with this positivity extending toward the central electrodes in the C9− group (Figure 3B). Statistical analysis revealed a significant group difference, primarily driven by a cluster of central electrodes, which appeared as a negative potential (Figure 3B). Further inspection of the average ERPs from these significant central electrodes confirmed that this difference was driven by an increased negative potential in the C9+ group (Figure 3C). The outcomes of the statistical models are provided in Table S7.

**FIGURE 4:**
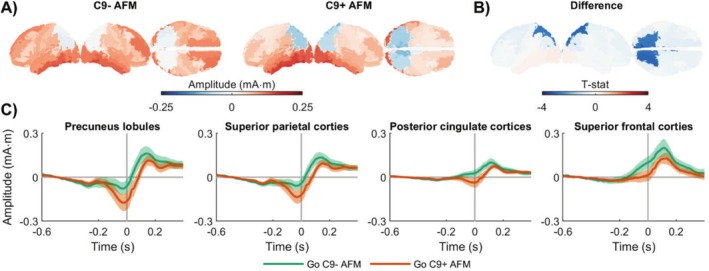
Differences in source‐space response‐locked brain activations in asymptomatic *C9orf72* family members. Group‐level response‐locked average Go potentials for each group, estimated from −100 to 100 ms around the response time. (B) A significant group difference was characterized by a more negative potential in the bilateral cuneus, precuneus, superior parietal, posterior midcingulate, and left isthmus cingulate regions in C9+ AFM. The significance initially detected in the superior frontal gyri did not remain after correction for multiple comparisons. (C) Average ERPs from these regions confirmed that this difference was driven by an increased negative potential in C9+ AFM in the posterior regions, whereas in the frontal regions, the signal appeared to be diminished in the C9+ AFM group. AFM, asymptomatic family members; C9−, carriership of *C9orf72* with normal repeat length; C9+, carriership of *C9orf72* repeat expansion.

The group‐level response‐locked Go ERPs in source‐space are shown in Figure 4A (see also Figure S4). Both groups exhibited activation in the middle frontal and inferior temporal cortices. Statistical analysis revealed a significant group difference in the bilateral cuneus, precuneus, superior parietal, posterior midcingulate, and left isthmus cingulate regions (Figure 4B). Similar to the stimulus‐locked findings, the significance initially detected in the superior frontal gyri did not remain after correction for multiple comparisons. Further inspection of the average ERPs from these regions confirmed that this difference was driven by an increased negative potential in the C9+ AFM group in the posterior regions, whereas in the frontal regions, the signal appeared to be diminished in the C9+ AFM group (Figure 4C). The outcomes of the statistical models are reported in Table S8.

### 3.7 ERP Group Differences Do Not Explain Similar SART Performance

To further explore the nature of these ERP differences and their relationship to intact SART performance, we conducted a linear model analysis. Based on the findings, we used the average stimulus‐locked N2 amplitude from each task condition separately, as well as the average response‐locked amplitude. For sensor‐space data, we used the average signal from central electrodes that showed significant differences, while for source‐space data, we used the average signal from the bilateral cuneus and superior parietal regions. These analyses did not reveal any significant relationships between ERP and SART after correction for multiple comparisons (Tables S9 and S10).

## 4 Discussion

This study demonstrates the potential of EEG to detect subtle alterations in cortical function among AFM carrying a pathological *C9orf72* RE. By comparing individuals with (C9+) and without (C9−) the mutation during the SART, a variation of a Go/NoGo task, we identified EEG differences specific to the *C9orf72* RE carriers in the asymptomatic phase. Notably, C9+ AFM exhibited a significantly increased negative potential at central electrodes around the time of the response in both NoGo and Go conditions. Source analysis localized these differences to the bilateral precuneus and superior parietal cortices. These findings suggest that the *C9orf72* RE may alter parietal network function, potentially contributing to the complex mechanisms underlying the increased risk of ALS and FTD. Our results underscore the value of EEG as a noninvasive, cost‐effective tool for detecting functional alterations in C9+ AFM, supporting its potential for further exploration of biomarkers for early ALS detection.

The cognitive mechanisms underlying response inhibition are often studied using Go/NoGo paradigms, which typically evoke a frontocentral negativity (N2 or N200 component) around 150–350 ms poststimulus, followed by a positive deflection (P3 or P300 component) spanning 250–600 ms. Both N2 and P3 are more pronounced in the NoGo condition than in the Go condition. While the precise functional roles and neural generators of these components remain incompletely understood, both are considered crucial for successful inhibition processes (Huster et al. 2013; Pires et al. 2014). The N2 is often interpreted as an index of response conflict and (pre‐)inhibition, with neural generators often linked with the medial prefrontal cortex, the midcingulate cortex, and presupplementary motor area, and potentially lateral frontal regions (Pires et al. 2014; Folstein and Van Petten 2008). The activity of the P3 is generally associated with response inhibition and performance monitoring, with its spatially distributed activity reflecting engagement of frontal and temporoparietal regions (Huster et al. 2013, 2020; Pires et al. 2014; Polich 2007). In our study, both the N2 and the P3 were evident across the frontoparietal axis, consistent with previous SART studies (McMackin et al. 2020; Staub et al. 2015; Zordan et al. 2008; Hart et al. 2012).

Our analysis revealed consistent differences in brain activation between C9− and C9+ AFM across both task conditions and in both stimulus‐ and response‐locked data. These differences occurred around the time of the motor response and were source‐localized to the bilateral precuneus and superior parietal cortices. While the frontal cortex is well known for its role in inhibition, growing evidence from various modalities suggests that the parietal cortex also plays a key role in motor control (Lindner 2018). Notably, stimulation of the superior parietal cortex has been shown to selectively inhibit voluntary hand movements, including their initiation (Desmurget et al. 2018). More broadly, the parietal cortex likely functions as a central hub where visual, cognitive, and motor‐related signals converge, integrating information to highlight behaviorally relevant stimuli and adaptively influence task‐dependent motor control (Freedman and Ibos 2018).

While this is the first study to investigate EEG differences in asymptomatic *C9orf72* RE carriers, we previously conducted an EEG study using the SART paradigm in ALS (McMackin et al. 2020). That study revealed increased activation in areas including the left inferior parietal cortex, which could align with the increased negative potential observed in the posterior brain regions here. However, as the previous study compared healthy controls with ALS patients, most of whom were C9−, those findings may not be directly applicable to the present study, which specifically examines changes associated with the *C9orf72* RE.

Our observation of altered EEG dynamics originating from parietal regions aligns with our recent longitudinal MRI study, which revealed both baseline cortical atrophy and accelerated cortical thinning in these areas in C9+ AFM (van Veenhuijzen et al. 2022). Additionally, a PET study has reported focal glucose hypermetabolism in the precuneus in C9+ AFM (De Vocht et al. 2020). While our EEG findings emphasize parietal alterations, studies have also reported grey matter atrophy (van Veenhuijzen et al. 2022; Bede et al. 2023) and glucose hypometabolism (De Vocht et al. 2020; Popuri et al. 2021) primarily in subcortical structures. The involvement of subcortical regions is further suggested by reports of reduced volumes in C9+ ALS patients compared to C9− ALS patients (Christidi et al. 2024). Although subcortical regions are likely implicated in motor control relevant to SART performance, reliable assessment of their activity with EEG is limited by their deep location and consequently, they were omitted from our source analysis.

Despite the observed involvement of the network implicated in motor control, none of the participants reported motor symptoms, which were assessed through a thorough physical examination at our outpatient clinic for neuromuscular disorders. Although some findings suggestive of mild upper motor neuron involvement, such as pseudobulbar reflexes and brisk tendon reflexes, were observed, they were present in both groups. These mild signs are known to occur in healthy individuals and do not necessarily indicate pathological changes (Dick 2003). Reflexes, however, may not be the most direct correlate for functions subserved by parietal regions, as they primarily assess corticospinal tract integrity, with major inputs from the primary motor cortex, brainstem, and spinal cord. Although frontal lobe regions can modulate some reflex responses, the core pathways for basic tendon jerks are distinct from the higher‐order sensorimotor integration of the parietal lobe.

Notably, SART performance measures did not differ between the two cohorts, and further analyses relating ERP measures to SART performance and *C9orf72* RE carriership revealed no significant associations after correction for multiple comparisons. This would suggest that the observed ERP differences may indicate an overly activated response of the parietal network without a clear pathological or compensatory role in SART performance. In fact, it may be that the SART is not sufficiently challenging and fails to engage the parietal cortex in a manner that reveals task performance deficits in C9+ AFM, resulting in a lack of clear correlates between the observed ERP differences and intact SART performance. Intriguingly, however, prior to multiple comparisons correction, ERP measures (i.e., the increased negativity) showed an association with the increased rate of anticipation errors primarily within the C9+ AFM cohort. This could suggest that *C9orf72* RE may subtly alter functional dynamics within the parietal network underlying response timing control and consequently cause some deficits in controlling premature responses. In contrast, the anticipation errors in the C9− group may arise from more heterogeneous sources not consistently linked to the parietal activity. While this group‐specific association, though exploratory, could potentially suggest a vulnerability of the parietal network linked to motor control and *C9orf72* RE carriership, the understanding of the observed EEG alterations could be improved by identifying correlates with specific tests of visuospatial and sensorimotor processing (Michielsen et al. 2025).

Some limitations of our study merit consideration. The nature of the observed differences remains unclear, as these findings could reflect neurodevelopmental differences, early compensatory mechanisms, or early pathological signs. Our recent MRI study of C9+ photoconverters to ALS/FTD revealed a pattern of regional vulnerability primarily confined to the frontotemporal and cingulate areas (van Veenhuijzen et al. 2025). Although group comparisons between C9+ and C9− AFM provide insight into mutation‐associated brain changes during the asymptomatic phase, they cannot determine whether such changes reflect imminent symptom onset or merely indicate broader disease susceptibility. Disentangling these possibilities and understanding their trajectory requires longitudinal studies, as our cross‐sectional design limits causal inferences. Additionally, we did not include a population‐based healthy control group, which restricts us from exploring alterations that may be present across these families regardless of the *C9orf72* RE status; however, comparing asymptomatic carriers to noncarriers within the same family provides a stringent statistical approach to isolate changes specifically associated with the *C9orf72* RE.

## 5 Conclusion

These findings provide the first evidence of functional EEG changes in asymptomatic *C9orf72* RE carriers. The observed changes in the parietal areas may stem from neurodevelopmental mechanisms or reflect disruptions in the regulation of the motor control network, potentially contributing to the early pathophysiology of the disease in some individuals. Given the promising nature of these changes, further longitudinal investigation is needed to clarify their potential as biomarkers for monitoring the risk of conversion to symptomatic disease.

## Conflicts of Interest

The authors declare no conflicts of interest.

## Supporting information


**Data S1.** Supporting Information.

## Data Availability

The data that support the findings of this study are available from the corresponding author, upon reasonable request from qualified investigators, after permission from the appropriate regulatory authorities.
