# Supplementary material for: Altered EEG Response of the Parietal Network in Asymptomatic C9orf72 Carriers
Source: Hum Brain Mapp. 2025 Jul 25;46(11):e70275. doi: 10.1002/hbm.70275 (PMC12290479; doi:10.1002/hbm.70275)
Supplement: Supplementary file 1 — Data S1. Supporting Information. [file HBM-46-e70275-s001.docx]

**Supplementary Note 1: Data preprocessing pipeline**

First, data were downsampled to 256 Hz, a highpass filter (non-causal Butterworth, 0.3 Hz cut-off, 4th order) was applied and line noise (at 50 and 100 Hz) was removed using the Zapline-plus algorithm.^1^

Bad electrodes were detected and removed using the PREP pipeline algorithm^2^ with the following thresholds: robust deviation threshold = 5, high-frequency noise threshold = 5, correlation threshold = 0.4, and bad time threshold = 0.03. Electrodes were further flagged for removal if the log-log spectral power slope between 7–45 Hz was positive for more than 50% of the recording time, indicating muscle activity contamination.^3^

After bad channel detection, affected electrodes were interpolated using spherical spline interpolation (see Supplementary Table 1).^4^ The data were then re-referenced to the common average. Independent component analysis (ICA) was performed using CUDAICA (GPU-optimised Infomax ICA),^5^ extracting 70 components using principle component analysis (retaining, on average, over 99% of the variance; see Supplementary Table 1).

Independent components (ICs) corresponding to ocular, muscle, and cardiac activity were identified using ICLabel (with a classification probability > 0.4 for eye and muscle activity),^6^ as well as correlations with bipolar vertical (R > 0.6) and horizontal (R > 0.4) ocular electrodes, and electrocardiogram (ECG) electrodes (R > 0.4). Additionally, cardiac-related ICs were identified using cross-trial phase statistics (Zscore(V) > 5, Pk > 20),^7^ while muscle-related ICs were identified as those with log-log spectral power slope between 7–45 Hz being positive. Ocular and cardiac ICs were completely removed, while muscle-related ICs were filtered (non-causal Butterworth, 15 Hz cut-off, 4th order) to preserve underlying low frequency neural activity.^8,9^ The number of artifact ICs was similar across the two cohorts (see Supplementary Table 1).

Cleaned signals were then lowpass filtered (non-causal Butterworth, 60 Hz cut-off, 4th order) and segmented into epochs ranging from -200 to 900 ms relative to stimulus event (stimulus-locked), and from -600 to 400 ms relative to response event (response-locked). Both datasets were then re-referenced to the common average and baseline-corrected by subtracting the mean amplitude of the baseline period: -200 to 0 ms for the stimulus-locked dataset, and -600 to -400 ms for the response-locked dataset. Epochs were then screened for residual artefacts and those exceeding ±75 μV were removed.

**Supplementary Table 1. Preprocessing outcomes and data quality assessments**

|  | **AFM C9-** | **AFM C9+** | **P-value** |
| --- | --- | --- | --- |
| **Preprocessing outcomes** |  |  |  |
| Interpolated electrodes | 6 (3-9) | 7 (5-12) | 0.12 |
| Variance explained with 70 ICs (%) | 99.4 (99.0-99.6) | 99.4 (98.6-99.6) | 0.54 |
| Artifact ICs | 6 (4-10) | 8 (5-11) | 0.13 |
| Analysed stimulus-locked NoGo/Go trials* | 62 (55-75) | 61 (52-76) | 0.77 |
| Analysed response-locked Go trials | 619 (568-668) | 626 (574-766) | 0.52 |
| **Sensor-space data quality** |  |  |  |
| Group-level dependability stimulus-locked NoGo^10^ | 0.98 | 0.98 | / |
| Group-level dependability stimulus-locked Go | 0.99 | 0.99 | / |
| Group-level dependability response-locked Go | 0.99 | 0.99 | / |

Data are shown as median (interquartile range). P-values were calculated using the Mann–Whitney U test. Abbreviations: AFM = asymptomatic family member; C9− = carriership of *C9orf72* with normal repeat length; C9+ = carriership of *C9orf72* repeat expansion; IC = independent component

^*^ The number of correct Go trials used in the analysis was matched per person to the number of correct NoGo trials.

**Supplementary Table 2. Outcomes of physical examination and cognitive screening**

|  | **AFM C9-** | **AFM C9+** | **P-value** |
| --- | --- | --- | --- |
| **Physical examination^a^** |  |  |  |
| Dysarthria | 0 (0) | 0 (0) | 1.00 |
| Impaired tongue movement | 0 (0) | 1 (3) | 1.00 |
| Sustained glabellar reflex | 1 (2) | 1 (3) | 0.85 |
| Jaw jerk reflex presence | 0 (0) | 2 (5) | 1.00 |
| Snout reflex presence | 7 (14) | 3 (8) | 0.31 |
| Palmomental reflex presence | 2 (4) | 6 (16) | 0.09 |
| Hypertonia arm muscles | 0 (0) | 0 (0) | 1.00 |
| Biceps tendon reflex   - Low-Normal - Brisk - Very brisk | 43 (86)  7 (14)  0 (0) | 28 (76)  9 (24)  0 (0) | 0.85 |
| Triceps tendon reflex   - Low-Normal - Brisk - Very brisk - Missing data | 42 (84)  7 (14)  0 (0)  1 (2) | 29 (78)  7 (19)  0 (0)  1 (3) | 0.36 |
| Deltoid tendon reflex presence | 9 (18) | 10 (27) | 0.30 |
| Trapezoid tendon reflex presence | 6 (12) | 6 (16) | 0.31 |
| Pectoral tendon reflex presence | 5 (10) | 5 (14) | 0.46 |
| Hoffmann’s reflex presence | 2 (4) | 3 (8) | 0.38 |
| Abdominal reflex absence | 4 (8) | 8 (22) | 0.09 |
| Hypertonia leg muscles | 0 (0) | 0 (0) | 1.00 |
| Knee jerk reflex   - Low-Normal - Brisk - Very brisk | 39 (78)  10 (20)  1 (2) | 29 (78)  6 (16)  2 (5) | 0.56 |
| Ankle jerk reflex   - Low-Normal - Brisk - Very brisk - Missing data | 43 (86)  6 (12)  1 (2)  0 (0) | 29 (78)  5 (14)  1 (3)  2 (5) | 0.44 |
| Adductor reflex presence | 11 (22) | 9 (24) | 0.54 |
| Plantar reflex Babinski response | 0 (0) | 0 (0) | 1.00 |
| **Cognitive screening (ECAS)^b^** |  |  |  |
| ALS specific | 86.5 (82.0-91.0) | 87.0 (82.0-91.0) | 0.90 |
| ALS nonspecific | 31.5 (30.0-33.0) | 31.0 (27.8-33.0) | 0.11 |
| Total | 118.0 (112.0-123.0) | 118.0 (111.5-122.0) | 0.61 |

The presence of the highest reflex or muscle tone, either on the left or right side, was used for each participant. Abbreviations: AFM = asymptomatic family member; C9− = carriership of *C9orf72* with normal repeat length; C9+ = carriership of *C9orf72* repeat expansion; ECAS = Edinburgh Cognitive and Behavioural ALS Screen; ALS = amyotrophic lateral sclerosis

**^a^** Data are shown in count (%). P-values were calculated using ordinal regression or binomial logistic regression for dichotomous outcomes, with assessment outcome as the response variable and age, sex, and *C9orf72* RE carriership as predictors.

**^b^** Data are shown as median (interquartile range). P-values were calculated using linear model analysis, with assessment outcome as the response variable and age, sex, education level, and *C9orf72* carriership as predictors.

**Supplementary Table 3. Linear mixed effects model outcomes for NoGo N2 significant electrodes**

| **Label** | **Difference (95% confidence interval)** | **P-value Uncorr** | **P-value Corr** |
| --- | --- | --- | --- |
| A1 | -1.81 (-3.08 - -0.53) | 0.006755 | 0.034593 |
| A13 | 2.33 (0.73 - 3.94) | 0.005536 | 0.046791 |
| A24 | 2.76 (1.04 - 4.48) | 0.002359 | 0.027594 |
| A25 | 2.74 (1.14 - 4.33) | 0.001166 | 0.018396 |
| A26 | 2.58 (1.01 - 4.16) | 0.001893 | 0.022396 |
| A27 | 2.89 (1.15 - 4.62) | 0.001634 | 0.019796 |
| A28 | 2.49 (0.78 - 4.20) | 0.005522 | 0.046791 |
| B1 | -1.57 (-2.71 - -0.42) | 0.008814 | 0.042991 |
| B7 | 2.56 (0.87 - 4.24) | 0.003869 | 0.039192 |
| B8 | 2.94 (1.23 - 4.66) | 0.001144 | 0.017596 |
| B9 | 2.97 (1.30 - 4.64) | 0.000797 | 0.014597 |
| B10 | 2.75 (1.47 - 4.04) | 0.000066 | 0.007798 |
| B11 | 2.11 (0.96 - 3.26) | 0.000553 | 0.012797 |
| B12 | 1.52 (0.62 - 2.43) | 0.001487 | 0.019796 |
| B20 | -1.45 (-2.39 - -0.50) | 0.003485 | 0.018996 |
| B21 | -1.16 (-1.97 - -0.35) | 0.006413 | 0.034593 |
| B30 | -1.13 (-1.97 - -0.30) | 0.009518 | 0.042991 |
| B31 | -1.43 (-2.28 - -0.57) | 0.001557 | 0.011598 |
| B32 | -1.81 (-2.74 - -0.87) | 0.000303 | 0.006399 |
| C1 | -1.91 (-3.05 - -0.77) | 0.001484 | 0.011598 |
| C2 | -2.01 (-3.13 - -0.90) | 0.000655 | 0.007399 |
| C3 | -1.77 (-2.76 - -0.78) | 0.000757 | 0.007399 |
| C11 | -1.89 (-2.97 - -0.81) | 0.000963 | 0.008598 |
| C12 | -1.65 (-2.76 - -0.55) | 0.004361 | 0.022396 |
| C21 | -1.51 (-2.60 - -0.42) | 0.008281 | 0.042991 |
| C22 | -1.75 (-2.87 - -0.62) | 0.003116 | 0.018996 |
| C23 | -2.12 (-3.26 - -0.97) | 0.000488 | 0.007399 |
| C24 | -1.79 (-2.83 - -0.74) | 0.001246 | 0.009798 |
| C25 | -1.44 (-2.48 - -0.39) | 0.008735 | 0.042991 |
| D1 | -2.01 (-3.18 - -0.84) | 0.001124 | 0.009798 |
| D2 | -1.86 (-2.97 - -0.75) | 0.001527 | 0.011598 |
| D12 | -1.27 (-2.14 - -0.41) | 0.004784 | 0.028394 |
| D13 | -1.50 (-2.38 - -0.61) | 0.001333 | 0.009798 |
| D14 | -1.50 (-2.53 - -0.48) | 0.005287 | 0.028394 |

**Supplementary Table 4. Linear mixed effects model outcomes for Go N2 significant electrodes**

| **Label** | **Difference (95% confidence interval)** | **P-value Uncorr** | **P-value Corr** |
| --- | --- | --- | --- |
| A1 | -1.51 (-2.43 - -0.60) | 0.001632 | 0.012597 |
| A2 | -1.19 (-1.95 - -0.42) | 0.003211 | 0.019996 |
| A23 | 2.15 (0.81 - 3.50) | 0.002365 | 0.031994 |
| A24 | 2.43 (0.99 - 3.86) | 0.001379 | 0.026995 |
| A25 | 2.32 (0.99 - 3.66) | 0.001009 | 0.024795 |
| A26 | 2.35 (0.96 - 3.74) | 0.001366 | 0.026995 |
| A27 | 2.53 (1.03 - 4.03) | 0.001359 | 0.026995 |
| A28 | 2.25 (0.76 - 3.74) | 0.003984 | 0.044191 |
| B1 | -1.29 (-2.09 - -0.50) | 0.002047 | 0.013997 |
| B2 | -0.86 (-1.49 - -0.23) | 0.008905 | 0.038392 |
| B9 | 2.15 (0.77 - 3.54) | 0.003128 | 0.035993 |
| B10 | 1.75 (0.65 - 2.85) | 0.002468 | 0.034193 |
| B20 | -1.18 (-1.87 - -0.48) | 0.001338 | 0.011798 |
| B21 | -0.94 (-1.55 - -0.34) | 0.002986 | 0.016797 |
| B22 | -0.69 (-1.22 - -0.17) | 0.011734 | 0.044991 |
| B30 | -0.83 (-1.46 - -0.20) | 0.011447 | 0.044991 |
| B31 | -0.94 (-1.64 - -0.24) | 0.010068 | 0.038392 |
| B32 | -1.26 (-1.98 - -0.53) | 0.001053 | 0.011398 |
| C1 | -1.55 (-2.45 - -0.66) | 0.000978 | 0.011398 |
| C2 | -1.43 (-2.29 - -0.56) | 0.001766 | 0.013997 |
| C3 | -1.10 (-1.90 - -0.31) | 0.007821 | 0.034793 |
| C11 | -1.19 (-2.07 - -0.31) | 0.009430 | 0.038392 |
| C23 | -1.42 (-2.36 - -0.49) | 0.003719 | 0.019996 |
| D1 | -1.50 (-2.41 - -0.59) | 0.001768 | 0.013997 |
| D2 | -1.29 (-2.13 - -0.44) | 0.003909 | 0.022595 |
| D12 | -0.93 (-1.56 - -0.31) | 0.004567 | 0.022595 |
| D13 | -1.01 (-1.66 - -0.35) | 0.003588 | 0.019996 |
| D14 | -1.16 (-1.90 - -0.42) | 0.002873 | 0.016797 |
| D15 | -1.33 (-2.16 - -0.49) | 0.002586 | 0.016797 |
| D18 | -0.87 (-1.48 - -0.26) | 0.006133 | 0.029794 |
| D19 | -0.70 (-1.22 - -0.18) | 0.009797 | 0.038392 |

**Supplementary Table 5. Linear mixed effects model outcomes for NoGo N2 significant brain regions**

| **Label** | **Difference**  **(95% confidence interval)** | **P-value**  **Uncorr** | **P-value**  **Corr** |
| --- | --- | --- | --- |
| lh-precuneus | -1.327e-04 (-2.181e-04 - -4.721e-05) | 0.003161 | 0.037792 |
| lh-superiorparietal | -1.160e-04 (-1.953e-04 - -3.659e-05) | 0.005321 | 0.045591 |
| rh-inferiorparietal | -1.177e-04 (-1.917e-04 - -4.368e-05) | 0.002517 | 0.032593 |
| rh-precuneus | -1.456e-04 (-2.429e-04 - -4.832e-05) | 0.004346 | 0.040992 |
| rh-superiorparietal | -1.277e-04 (-2.025e-04 - -5.295e-05) | 0.001235 | 0.027395 |

**Supplementary Table 6. Linear mixed effects model outcomes for Go N2 significant brain regions**

| **Label** | **Difference**  **(95% confidence interval)** | **P-value**  **Uncorr** | **P-value**  **Corr** |
| --- | --- | --- | --- |
| lh-cuneus | -5.764e-05 (-9.365e-05 - -2.163e-05) | 0.002365 | 0.025795 |
| lh-precuneus | -1.294e-04 (-2.039e-04 - -5.492e-05) | 0.001027 | 0.019596 |
| lh-superiorparietal | -1.175e-04 (-1.825e-04 - -5.238e-05) | 0.000665 | 0.016797 |
| rh-inferiorparietal | -1.096e-04 (-1.766e-04 - -4.263e-05) | 0.001911 | 0.024595 |
| rh-precuneus | -1.261e-04 (-2.107e-04 - -4.160e-05) | 0.004454 | 0.039992 |
| rh-superiorparietal | -9.505e-05 (-1.551e-04 - -3.500e-05) | 0.002652 | 0.025795 |

**Supplementary Table 7. Linear mixed effects model outcomes for response-locked Go significant electrodes**

| **Label** | **Difference (95% confidence interval)** | **P-value Uncorr** | **P-value Corr** |
| --- | --- | --- | --- |
| A1 | -1.39 (-2.19 - -0.58) | 0.001103 | 0.015597 |
| A2 | -1.03 (-1.67 - -0.39) | 0.002405 | 0.019396 |
| A13 | 1.76 (0.58 - 2.94) | 0.004516 | 0.045791 |
| A23 | 1.91 (0.64 - 3.17) | 0.004111 | 0.045791 |
| A24 | 2.12 (0.80 - 3.43) | 0.002197 | 0.035193 |
| A25 | 2.06 (0.87 - 3.24) | 0.001012 | 0.026595 |
| A26 | 2.10 (0.88 - 3.32) | 0.001105 | 0.027794 |
| A27 | 2.20 (0.85 - 3.54) | 0.001982 | 0.031594 |
| B1 | -1.14 (-1.82 - -0.46) | 0.001392 | 0.015997 |
| B2 | -0.70 (-1.21 - -0.20) | 0.007999 | 0.038992 |
| B9 | 1.86 (0.65 - 3.07) | 0.003329 | 0.041592 |
| B10 | 1.58 (0.58 - 2.58) | 0.002647 | 0.039992 |
| B20 | -1.08 (-1.70 - -0.47) | 0.000864 | 0.013597 |
| B21 | -0.85 (-1.40 - -0.30) | 0.003309 | 0.023795 |
| B31 | -0.90 (-1.56 - -0.24) | 0.008660 | 0.044391 |
| B32 | -1.14 (-1.83 - -0.45) | 0.001670 | 0.017796 |
| C1 | -1.47 (-2.29 - -0.65) | 0.000736 | 0.012997 |
| C2 | -1.38 (-2.19 - -0.58) | 0.001171 | 0.015597 |
| C11 | -1.17 (-2.01 - -0.33) | 0.007859 | 0.038992 |
| C23 | -1.34 (-2.22 - -0.46) | 0.003866 | 0.026195 |
| D1 | -1.39 (-2.23 - -0.55) | 0.001672 | 0.017796 |
| D2 | -1.23 (-2.02 - -0.44) | 0.003076 | 0.023795 |
| D14 | -1.01 (-1.66 - -0.37) | 0.002803 | 0.020996 |
| D15 | -1.16 (-1.89 - -0.44) | 0.002317 | 0.019396 |
| D16 | -0.79 (-1.37 - -0.20) | 0.009957 | 0.044391 |

**Supplementary Table 8. Linear mixed effects model outcomes for response-locked Go significant brain regions**

| **Label** | **Difference**  **(95% confidence interval)** | **P-value**  **Uncorr** | **P-value**  **Corr** |
| --- | --- | --- | --- |
| lh-cuneus | -4.591e-05 (-7.373e-05 - -1.810e-05) | 0.001747 | 0.023595 |
| lh-isthmuscingulate | -4.942e-05 (-8.407e-05 - -1.476e-05) | 0.006445 | 0.040592 |
| lh-posteriorcingulate | -4.316e-05 (-7.115e-05 - -1.517e-05) | 0.003334 | 0.027195 |
| lh-precuneus | -8.915e-05 (-1.505e-04 - -2.781e-05) | 0.005538 | 0.033593 |
| lh-superiorparietal | -7.222e-05 (-1.242e-04 - -2.020e-05) | 0.007928 | 0.047990 |
| rh-cuneus | -4.071e-05 (-6.963e-05 - -1.180e-05) | 0.007128 | 0.040592 |
| rh-posteriorcingulate | -5.333e-05 (-8.925e-05 - -1.742e-05) | 0.004633 | 0.030794 |
| rh-precuneus | -1.015e-04 (-1.702e-04 - -3.279e-05) | 0.004843 | 0.030194 |
| rh-superiorparietal | -7.578e-05 (-1.252e-04 - -2.638e-05) | 0.003493 | 0.029194 |

**Supplementary Table 9. ERP-SART relationship: Entire sample associations**

|  | Stimulus-locked N2 | | | | Response-locked | |
| --- | --- | --- | --- | --- | --- | --- |
|  | Sensor  NoGo | Sensor  Go | Source  NoGo | Source  Go | Sensor  Go | Source  Go |
| Go correct | / | T = 1.24,  P = 0.21 | / | T = 0.11,  P = 0.91 | T = 1.75,  P = 0.08 | T = 0.93,  P = 0.35 |
| NoGo correct | T = 0.95,  P = 0.34 | / | T = 0.67,  P = 0.50 | / | / | / |
| Anticipation error | T = -1.07,  P = 0.29 | T = -0.4,  P = 0.69 | T = -0.81,  P = 0.42 | T = -0.82,  P = 0.41 | T = -1.69,  P = 0.09 | T = -1.75,  P = 0.08 |
| Response time | T = -1.00,  P = 0.32 | T = -1.74,  P = 0.08 | T = -0.29,  P = 0.77 | T = -0.59,  P = 0.56 | T = -0.44,  P = 0.66 | T = 0.30,  P = 0.76 |
| Response time  variability | T = -0.56,  P = 0.58 | T = -1.61,  P = 0.11 | T = 0.50,  P = 0.62 | T = -0.37,  P = 0.71 | T = -0.31,  P = 0.76 | T = 0.58,  P = 0.56 |

Data shown are T-statistics and associated P-values for the SART predictor variable from the linear mixed-effects model: EEG ~ AGE + SEX + GROUP + SART + (1|ID) + (1|PED). This model assumes that the relationship between ERP and SART is the same for both groups (i.e., regardless of the *C9orf72* status). P-values are not corrected for multiple comparisons.

**Supplementary Table 10. ERP-SART relationship: Group-specific associations**

|  | Stimulus-locked N2 | | | | Response-locked | |
| --- | --- | --- | --- | --- | --- | --- |
|  | Sensor  NoGo | Sensor  Go | Source  NoGo | Source  Go | Sensor  Go | Source  Go |
| Go correct | / | T = 0.47,  P = 0.64 | / | T = -0.05,  P = 0.96 | T = 0.34,  P = 0.74 | T = 0.56,  P = 0.57 |
| NoGo correct | T = 0.71,  P = 0.48 | / | T = 1.66,  P = 0.10 | / | / | / |
| Anticipation error | T = -2.18,  P = 0.03 | T = -1.83,  P = 0.07 | T = -2.33,  P = 0.02 | T = -2.14,  P = 0.03 | T = -1.93,  P = 0.05 | T = -2.41,  P = 0.02 |
| Response time | T = 1.18,  P = 0.24 | T = 1.11,  P = 0.27 | T = 1.10,  P = 0.27 | T = 1.08,  P = 0.28 | T = 0.93,  P = 0.35 | T = 0.82,  P = 0.41 |
| Response time  variability | T = 0.85,  P = 0.39 | T = 0.71,  P = 0.48 | T = 0.61,  P = 0.54 | T = 0.66,  P = 0.51 | T = 0.48,  P = 0.63 | T = 0.04,  P = 0.97 |

Data shown are T-statistics and associated P-values for the interaction term (GROUP_AFMC9+:SART) from the linear mixed-effects model: EEG ~ AGE + SEX + GROUP*SART + (1|ID) + (1|PED). This model allows the relationship between ERP and SART to vary by group (i.e., *C9orf72* status), thereby enabling the identification of potential group-specific dynamics, such as distinct compensatory mechanisms that might be evident in the cohort with the *C9orf72* repeat expansion. Relationships between ERP and SART for AFM C9- were not significant (data not shown). P-values are not corrected for multiple comparisons. Abbreviations: AFM = asymptomatic family member; C9− = carriership of *C9orf72* with normal repeat length; C9+ = carriership of *C9orf72* repeat expansion

**
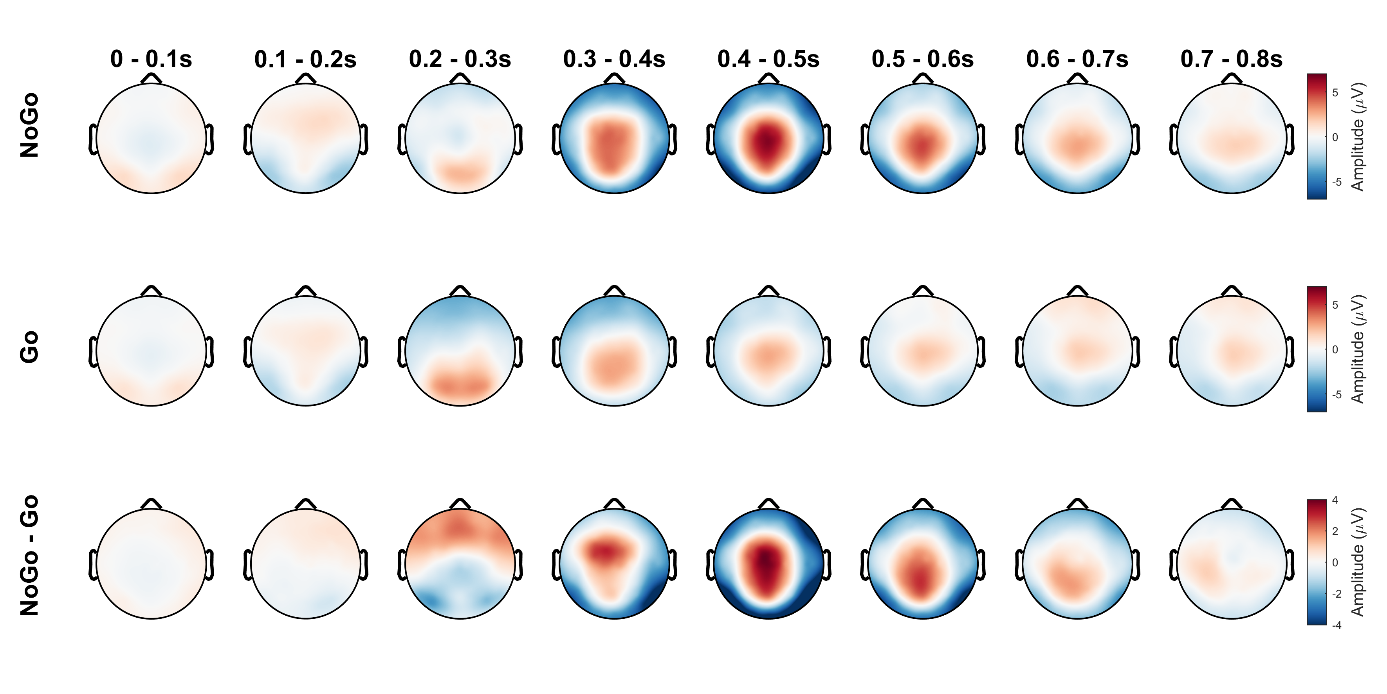
Supplementary Figure 1. The group-level (N = 87) sensor-space stimulus-locked activations of each task condition and the difference between the two conditions.**


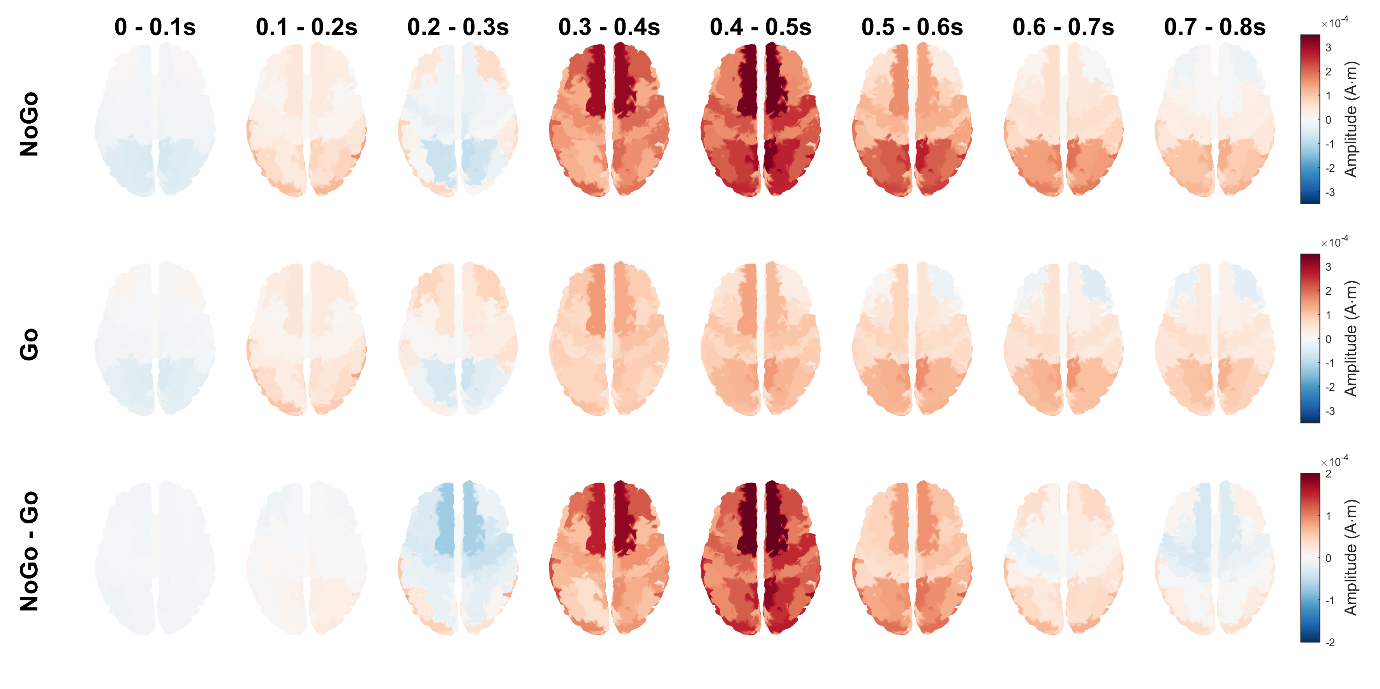


S**upplementary Figure 2. The group-level (N = 87) source-space stimulus-locked activations of each task condition and the difference between the two conditions.**

**
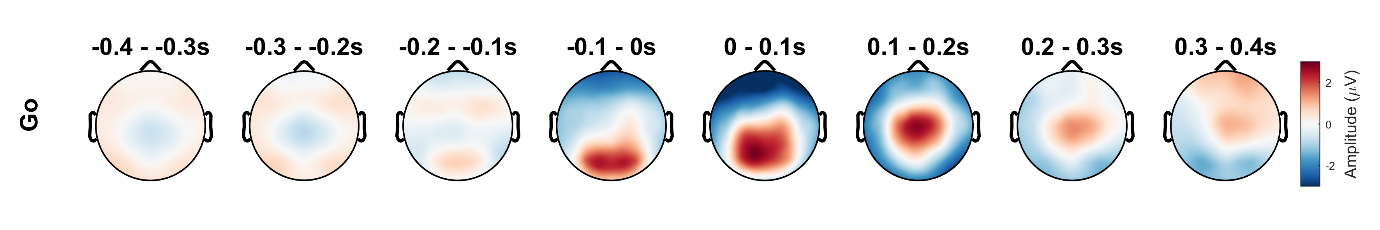
Supplementary Figure 3. The group-level (N = 87) sensor-space response-locked activations of the Go condition.**

**
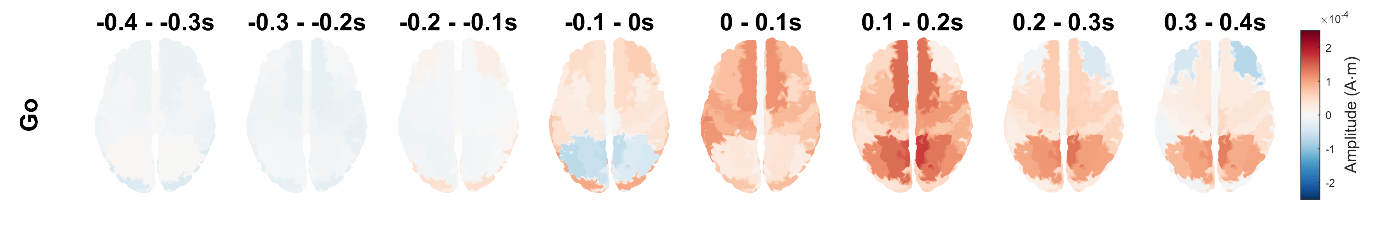
**

**Supplementary Figure 4. The group-level (N = 87) source-space response-locked activations of the Go condition.**

# References

1. Klug, M. & Kloosterman, N. A. Zapline-plus: A Zapline extension for automatic and adaptive removal of frequency-specific noise artifacts in M/EEG. *Hum Brain Mapp* **43**, 2743–2758 (2022).

2. Bigdely-Shamlo, N., Mullen, T., Kothe, C., Su, K. M. & Robbins, K. A. The PREP pipeline: Standardized preprocessing for large-scale EEG analysis. *Front Neuroinform* **9**, 1–19 (2015).

3. Dharmaprani, D. *et al.* A comparison of independent component analysis algorithms and measures to discriminate between EEG and artifact components. *Proceedings of the Annual International Conference of the IEEE Engineering in Medicine and Biology Society, EMBS* **2016-October**, 825–828 (2016).

4. Perrin, F., Pernier, J., Bertrand, O. & Echallier, J. F. Spherical splines for scalp potential and current density mapping. *Electroencephalogr Clin Neurophysiol* **72**, 184–7 (1989).

5. Raimondo, F., Kamienkowski, J. E., Sigman, M. & Fernandez Slezak, D. CUDAICA: GPU optimization of infomax-ICA EEG analysis. *Comput Intell Neurosci* **2012**, (2012).

6. Pion-Tonachini, L., Kreutz-Delgado, K. & Makeig, S. ICLabel: An automated electroencephalographic independent component classifier, dataset, and website. *Neuroimage* **198**, 181–197 (2019).

7. Dammers, J. *et al.* Integration of amplitude and phase statistics for complete artifact removal in independent components of neuromagnetic recordings. *IEEE Trans Biomed Eng* **55**, 2353–2362 (2008).

8. Bailey, N. W. *et al.* RELAX part 2: A fully automated EEG data cleaning algorithm that is applicable to Event-Related-Potentials. *Clinical Neurophysiology* **149**, 202–222 (2023).

9. Bailey, N. W. *et al.* Introducing RELAX: An automated pre-processing pipeline for cleaning EEG data - Part 1: Algorithm and application to oscillations. *Clinical Neurophysiology* **149**, 178–201 (2023).

10. Clayson, P. E., Brush, C. J. & Hajcak, G. Data quality and reliability metrics for event-related potentials (ERPs): The utility of subject-level reliability. *International Journal of Psychophysiology* **165**, 121–136 (2021).
